# Multi-cell, non-invasive, online monitoring of PLA-coated magnetic nanoparticles uptake by MCF-7 cells using digital holographic microscopy

**DOI:** 10.1016/j.bioactmat.2026.03.053

**Published:** 2026-04-16

**Authors:** Joel Kuhn, Nick W. Johnson, Christian Curcillo, Lewis A. MacGillivray, Leonard J. Nelson, Boris Polyak, Pierre O. Bagnaninchi, Humphrey H.P. Yiu

**Affiliations:** a Institute of Chemical Sciences, School of Engineering and Physical Sciences, Heriot-Watt University, Scotland, Edinburgh, EH14 4AS, UK; b Centre for Biomedicine & Global Health, School of Applied Sciences, Sighthill Campus, Edinburgh Napier University, Sighthill Court, Scotland, Edinburgh, EH11 4BN, UK; c Gold Standard Diagnostics Horsham LLC, 795 Horsham Rd, Horsham, PA, 19044, USA; d Centre for Regenerative Medicine. Institute for Regeneration and Repair, The University of Edinburgh, Edinburgh BioQuarter, 5 Little France Drive, Edinburgh, EH16 4UU, UK

**Keywords:** Digital holographic microscopy, Roughness kurtosis, MCF-7 breast cancer cells, PLA-coated magnetic particles, PEG-coated nanoparticles

## Abstract

The application of nanoparticles (NPs) in medicine as delivery platforms has experienced a significant expansion in the past decade and as a result, various imaging techniques have been applied to monitor cellular NP uptake. Although, high-resolution images of a NP contained within the cell can be generated allowing for quantification, live NP monitoring remains a challenge. Digital holographic microscopy (DHM) has emerged a convenient label-free method to study the cell surface dynamics in real-time by recording a hologram of an interference pattern generated by light which has been scattered from the cell sample, compared to the reference sample. Herein, we report that DHM can be used to monitor the cellular NP uptake in real-time by analysing the changing in surface roughness. As a proof-of-concept, we demonstrated an increase in cell roughness of MCF-7 human breat cancer cells when PLA-coated magnetite, Fe_3_O_4_ NPs interacted with the MCF-7 cell surface. By measuring cell roughness every minute, the entire NP internalisation process could be monitored. DHM also addressed challenges within existing imaging techniques, by enabling multi-cellular analysis as well as successfully monitor how changes in NP density and size impacted internalisation rate, highlighting how this method can be further applied in monitoring cellular dynamics and in the development of future nanoparticle-based therapeutics, as well as, tissue engineering.

## Highlights

• Uptake of magnetic nanoparticles (NPs) by MCF-7 breast cancer cells was monitored by Digital holographic microscopy (DHM).
• The NP uptake process was monitored based on the change of roughness on the cell surface induced by loading NPs.
• The effect of the density of NPs on uptake was small, while the particle size showed a more significant effect.
• Roughness kurtosis mode was more sensitive for uptake monitoring than roughness skewness.
• The technique was not size-limited as smaller NPs (30 nm in diameter) have shown a faster uptake.

## Graphical abstract

**Figure:**
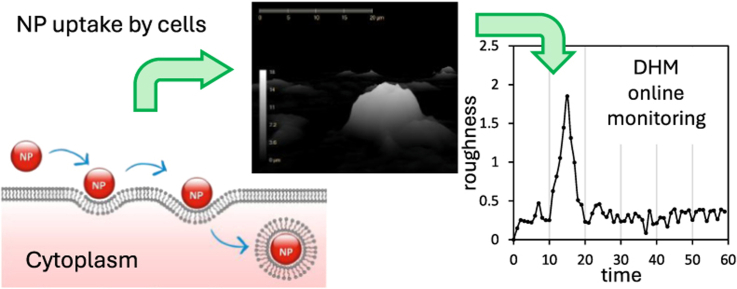

## 1 Introduction

The use of nanoparticles (NPs) in medical applications is a widely researched area [[1], [2], [3], [4], [5]], including within diagnostics [6,7], drug delivery [[8], [9], [10]], gene therapy [11,12], as well as hyperthermia treatments [13,14]. Each NP destined for medical use requires a toxicological assessment to verify safety when used in humans and to this end, a critical understanding of the mode of action is required [15,16]. In this instance, the interaction between NP and the targeted cell, as well as subsequent cellular uptake (or internalisation), needs to be realised to determine uptake efficiency in addition to intracellular NP concentration [17,18]. Research on magnetic nanoparticles (MNPs) is a major branch of nanomaterials research due to their additional physical property, magnetism, which offers various advantages to many applications, notably those in biomedicine [2,4,13,14]. Studying the uptake of MNPs by cells will be of high relevance to biomedical nanotechnology.

The mosaic model of a cell membrane describes the phospholipid bilayer as dynamic and fluidised which mediates the flow of materials and nutrients in and out of the cell [19,20]. NPs, however, cannot passively cross cell membranes by concentration gradient alone, and active transport mechanisms are required for their uptake [21]. The principal mechanism of cellular NP uptake is through endocytosis, although this process is governed by the cell and depends on a multitude of factors, including NP size and morphology [22]. During endocytosis, the cell membrane deforms through invagination, forming a cavity at the exterior of the cell, NP(s) then enter this new cavity where the cell membrane then buds off inside the cell forming an endosome [22].

Monitoring NP cellular uptake currently relies on chemical analysis [23,24], flow cytometry [[25], [26], [27]], fluorescence [[28], [29], [30]], and microscopy [31]. Flow cytometry allows for the high-throughput analysis of live cells and has been applied to track cellular internalisation of NPs when coupled with another imaging techniques [32,33]. Advancements in electron microscopy (EM) have enabled high-resolution cellular images, facilitating the analysis of cellular structure, but EM is not conducive to imaging live cells [[34], [35], [36]]. Both flow cytometry and EM cannot monitor cellular NP uptake in real time. Similarly, although atomic force microscopy (AFM) has been demonstrated to monitor cellular surface interactions, the techniques require the sample cells to be dried prior to imaging [37,38]. Conversely, numerous optical microscopy techniques are used to capture images of live cells and to monitor NP cellular uptake. However, cells do not absorb visible light and appear transparent under an optical microscope; therefore, contrast agents are required. Confocal laser scanning microscopy (CLSM), for example, relies on cell staining, this can damage the cell surface and interfere with the real-time analysis of NP cellular uptake [[39], [40], [41], [42]]. Fluorescent probes are another contrast agent commonly used conjunction with NPs, but many NPs are chemically inert, and if functionalised, surface modification can affect the cellular internalisation process [43]. Additionally, optical imaging techniques suffer from low frame rates and poor nanoscale resolution (e.g., 1/40th of a second to acquire a 512 × 512-pixel image), hindering the ability to monitor cellular NP uptake in real time and often missing the internalisation process entirely [44].

Digital holographic microscopy (DHM) is an alternative optical microscopy technique that combines digital holography with microscopy and is a non-invasive, label-free method which can provide high-resolution quantitative phase images of live cells [45,46]. A digital hologram records the interference pattern produced by light waves transmitted through or reflected from the specimen, rather than forming a direct optical image as in conventional microscopy [46]. Generally, reflection DHM is used to analyse reflective surfaces, whereas transmission DHM is best suited for transparent or semi-transparent specimens, including living cells [47]. In transmission DHM, an object beam passes through the specimen and a microscope objective, which magnifies the transmitted wave and minimises pixel-size limitations in the reconstructed image [48]. The transmitted light then meets a reference beam at a beam splitter, where the two coherent waves interfere to produce a hologram recorded by a CCD camera. An interference pattern encodes the optical path differences between the object and reference beams, enabling quantitative phase reconstruction and real-time visualisation of living cells in standard culture vessels [49,50]. This allows living cells to be visualised directly in petri dishes, as well as on microscopic slides, in real time, circumventing lengthy sample preparations and enabling the study of cell dynamics and responses to external stimuli. DHM has subsequently been used extensively for medical imaging as a simple method to study human cell morphology [[51], [52], [53]]. For example, Kemper et al. used transmission DHM to study dynamic cellular processes upon introduction of exogenous compounds into pancreatic cancer cells. The researchers were able to monitor changes in cell shape and quantify changes in membrane thickness in PaTu 8988T and PaTu 8988T pLXIN E-Cadherin cancer cells upon addition of the marine toxin Latrunculin B, laying the foundation for drug-induced cell dynamics monitoring [54]. DHM has also been applied to phenotypic screening for small-molecule drug discovery, and Farzam Rad et al. used transmission DHM to study the effect of calcium on the topography of human red blood cells. In this study, changes in cell contour roughness were monitored, with the researchers demonstrating that calcium-induced hydrophobic phospholipid aggregates form at the cell surface and highlighting how further DHM studies could enable a better understanding of the effect of hypercalcemia at the single-cell level [55]. Beyond single cell analysis, Dubois et al. tracked the migration and quantitatively analysed multiple HT-1080 fibrosarcoma cells cancer cells *in vitro* using DHM, highlighting the application of the technique in both multi-cell monitoring as well as, more broadly, in pathological diagnostics [56].

**Fig. 1:**
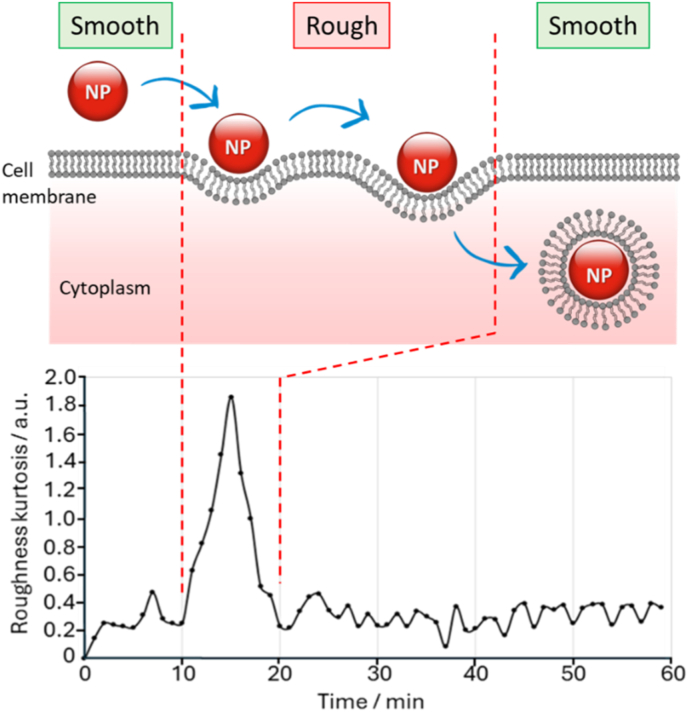
Conceptual image illustrating endocytosis of nanoparticles and the surface roughness generated that is to be monitored by DHM.

To this end, we hypothesised that transmission DHM could be applied to the real-time monitoring and quantification of cellular uptake of NPs in human cells, reducing the sample preparation complexity in current methods. As shown in Fig. 1, cellular-NP interaction can be measured by monitoring cell surface roughness, with NP adsorption inducing an increase in roughness. Since the DHM inherent frame time is shorter than most other comparable techniques, DHM would allow for real-time monitoring and potentially acquiring valuable information regarding the uptake mechanism [57]. The technique is non-invasive and requires no additional labelling, therefore, providing a more accurate representation of the uptake process. Composite magnetite, Fe_3_O_4_, nanoparticles were chosen as the model NPs because of their compatible size and tunable density and were tested alongside the human breast cancer cell line, MCF-7. Various functionalised NPs have been demonstrated to inhibit growth and cause apoptosis of this cell line, so an effective quantitative method to monitor the cellular NP uptake is of importance [58,59]. In particular, we compared two operational modes of the DHM, kurtosis and skewness, to monitor the cell contour roughness after incubating the NPs with the cell line. In this work, we have chosen two series of NPs as model NP samples; PLA-coated magnetic nanoparticles (MP-nX series) and 30 nm Fe_3_O_4_ magnetic nanoparticles. Both series have been used in our previous studies and were successfully internalised in primary neural stem cells (NSC) [60] and HepaRG cells [61], respectively.

## 2 Materials and methods

### 2.1 Materials

All reagents and solvents were bought from commercial sources without further purification, unless specified. All solvents were supplied by Fisher Scientific (Pittsburgh, PA, USA) and were of HPLC grade. Poly(D,L-lactide) (average M_w_: 75–120 kDa), ferric chloride hexahydrate, ferrous chloride tetrahydrate, sodium hydroxide, oleic acid, and PVA (87-90% hydrolysed, average Mw: 30-70 kDa), were purchased from Sigma-Aldrich™. Fe_3_O_4_ NPs (Nanopowder, 30 nm) were purchased from Alfa Aesar. PEG-silane, 3-[methoxy(polyethyleneoxy)_9-12_]propyltrimethoxysilane (Tech. grade, Mw: 591-719) was purchased from Fluorochem, UK. Poly(D,L-lactide) covalently labelled with BODIPY® 564/570 (Life Technologies™) was a generous gift to BP from Dr. Robert Levy, Children's Hospital of Philadelphia, U.S.A with the synthesis procedure and characterisation reported in Ref. [62]. Distilled water used in all experimental procedures was obtained using a Milli-Q water purification system.

### 2.2 Preparation for PLA-coated MP-0X to MP-5X particles

The NP samples used in this study were the PLA-coated magnetic magnetite, Fe_3_O_4_, NPs and denoted as MP-1X, MP-3X and MP-5X, according to their magnetite content, with the sample labelled MP-0X containing no magnetite. Preparation has been reported in previous studies in a multi-step procedure [60,63,64]. In summary, the Fe_3_O_4_ NPs were prepared by co-precipitation, based on the Massart method, using FeCl_2_ and FeCl_3_ as the iron precursors and NaOH as a base [65]. The resultant Fe_3_O_4_ nanoparticles were then coated with oleic acid and dispersed in chloroform to form a stable magnetic fluid. PLA was then emulsified in a mixture of magnetic fluid and an aqueous solution of polyvinyl alcohol (PVA) by sonication. After removing the organic solvent under reduced pressure, the nanoparticles were then lyophilised in trehalose (aq., 10% w/v) and stored at 4 °C before use. A more detailed method used to prepare the magnetic nanoparticles can be found in Section 1.1. in the Supplementary Information.

### 2.3 Preparation for PEG-coated 30 nm NPs

PEG-coated magnetite nanoparticles were prepared using a silanisation method following the procedure reported in Ref. [61]. Fe_3_O_4_ (500 mg, 2.16 mmo, 2.8 equiv.) NPs (30 nm) were dispersed in anhydrous toluene (50 mL) using an ultrasonic water bath (Advantage Lab, AL-04-04, 80W) at full power for 15 min before 3-[methoxy(polyethyleneoxy)_9-12_]propyltrimethoxysilane (0.5 mL, ca. 0.763 mmol, 1 equiv.) was added to the suspension and stirred under reflux. After 6 h, the resultant PEG-coated Fe_3_O_4_ NPs were recovered using a NdFeB magnet, washed with acetone (10× 5 mL), and dried at 50 °C under reduced pressure.

### 2.4 Characterisation of NPs

NPs were fully characterized using transmission electron microscopy (TEM), Fourier-transform infrared (FTIR) spectroscopy, solid-state magic angle spinning nuclear magnetic resonance (MAS NMR) spectroscopy, magnetometry, zeta potential measurement, elemental analysis, and dynamic light scattering (DLS), with the full experimental procedures contained in Section 1.2 in the Supplementary Information.

### 2.5 Cell culture

#### 2.5.1 General

MCF-7 human breast adenocarcinoma cells were obtained from the American Type Culture Collection (ATCC, USA). All apparatus used were either sterile from suppliers or autoclaved prior to use.

#### 2.5.2 Preparation of cell culture medium I

For 1 L media: Dulbecco's modified eagles' medium (880 mL), foetal bovine serum (FBS) (100 mL) and L-glutamine (10 mL, aq., 200 mM) were mixed in a volumetric flask and topped up to 1 L using dH_2_O. The media was then transferred to a duran flask, autoclaved at 121 °C for 20 min and stored at 4 °C. For the serum-free medium, FBS was omitted.

#### 2.5.3 Culturing conditions of MCF-7 breast cancer cells

Unless stated otherwise, MCF-7 breast cancer cells were routinely cultured in Medium I containing penicillin/streptomycin (1:1, 1 % w/v), using a sterile T25 cell culture flask (5% CO_2_ atmosphere, BOC, UK), at 37 °C. For MCF-7 cell cultures, culture medium was changed every two days; cells were routinely split at 80%-90% confluency. Cells were seeded at 80,000 cells per dish (see below).

### 2.6 Microscopy

#### 2.6.1 DHM monitoring

On a sterile Petri dish (Ø 25 mm), medium I (2 mL) was inoculated with MCF-7 cancer cells (final concentration = 1 x 10^5^ cells) and incubated at 37 °C in a 5% CO_2_ atmosphere. After 48 h, the medium was replaced with serum-free medium (2 mL) supplemented with a NP (150 μL, 5 μg/mL) suspension. The morphology of cells after NP loading was digitally monitored using a HoloMonitor M4 time lap microscope (Phase Holographic Imaging, Sweden). Cells loaded with NPs were incubated at 37 °C and 5% CO_2_ atmosphere directly on the DHM stage. The frame capture rate was set to take an image every minute, with a total imaged area of 1.558 mm^2^. The captured frames generated a time-lapse video showing cell proliferation. After automatic segmentation of each cell in the field of view, several parameters derived from the optical phase and its spatial and temporal derivatives were calculated for each single cell. Surface roughness parameters, including skewness and kurtosis (see also Fig. S1), were derived from changes in the optical path within the region of interest. After NP loading and DHM measurement, the cell culture medium was replaced with medium I (containing serum) for further use. Each experiment was performed in duplicate.

#### 2.6.2 DHM data analysis

The data acquired from the DHM experiments were analysed using the HStudio program. “Cell kurtosis” and “cell skewness” were plotted against time for each segmented cell, with cells not showing a significant peak for kurtosis or skewness, respectively, being rejected. The plots were normalised for data analysis to align the peak start times, as the time prior is likely the time taken for the NP to travel to the cell membrane and is determined to be irrelevant for the purposes of this study. Additionally, cells which exhibited a peak at t = 0 min and cell death prior to NP cellular uptake were discarded. A detailed step-by-step procedure can be found in the Supplementary Information (see also Fig. S2).

Quantitatively, NP uptake was inferred from time-resolved changes in DHM-derived cell-surface roughness descriptors (kurtosis and skewness, as calculated in HStudio). NP interaction with the cell produced a transient increase in apparent roughness, observed as a kurtosis peak, followed by a return toward baseline as internalisation proceeded. This apparent roughening is consistent with local changes in optical path length caused by refractive-index contrast between the nanoparticle and the surrounding cell material.

## 3 Results

### 3.1 NP characterisation

The study began with the synthesis and characterisation of the NPs required for DHM analysis. Fe_3_O_4_ NPs were chosen due to their biocompatibility [64,66], size [67], and tuneable density [60,68] and were synthesised by co-precipitation [60]. The magnetic responsiveness of the NPs can be tuned by modulating the amount of incorporated magnetite within a polymeric coating, without significantly affecting particle size or surface charge [60]. As a result, a series of PLA-coated Fe_3_O_4_ NPs were prepared (MP-0X, MP-1X, MP-3X, MP-5X) using a modified emulsification-solvent evaporation method to enable comparative analysis of how different levels of iron oxide content impact cellular uptake, as well as to control particle size and reduce particle agglomeration. The PLA coating was also doped with PLA labelled with BODIPY® (9% w/w) to enable fluorescent image analysis. Smaller polyethylene glycol (PEG)-coated Fe_3_O_4_ NPs (30 nm) were also synthesised for two reasons: (i) a previous cell internalisation study demonstrated that the PEG coating induced a reduction in NP aggregation, (ii) to perform comparative NP analysis relating to differences in NP size [61].

**Fig. 2:**
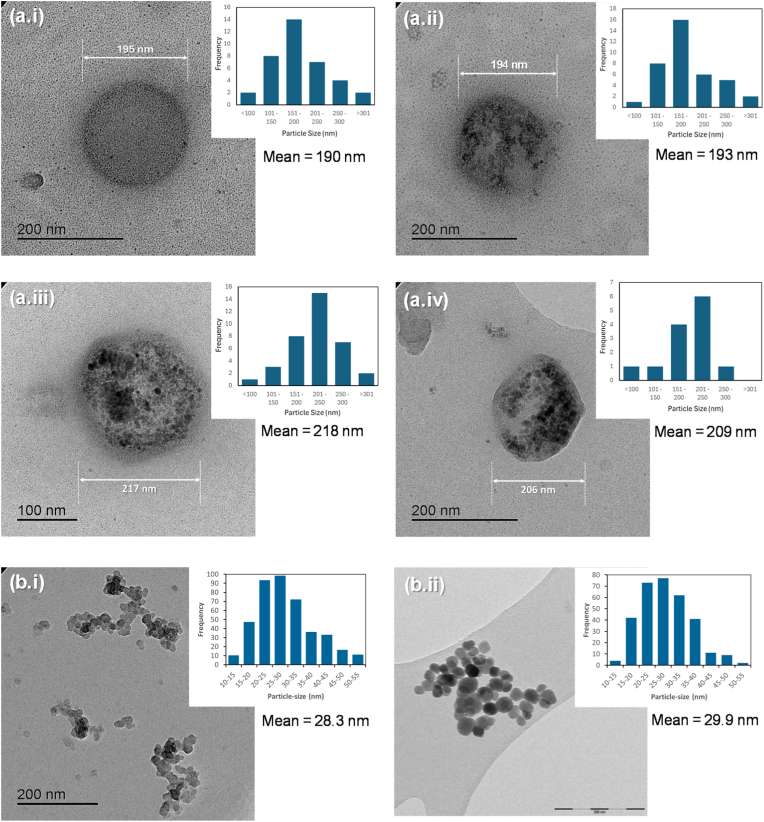
TEM images of (a) PLA-coated NP samples and (b) Fe_3_O_4_ 30 nm NP samples; (a.i) MP-0X, (a.ii) MP-1X, (a.iii) MP-3X, (a.iv) MP-5X, (b.i) unfunctionalised Fe_3_O_4_ 30 nm NPs, (b.ii) PEG_9-12_-Fe_3_O_4_ 30 nm NPs. The particle size distribution of each sample is shown on the inset.

After fabrication, characterisation using TEM revealed that the prepared NPs have a spherical to “pseudo-spherical” morphology with a mean particle diameter ranging from 190 to 218 nm (Fig. 2ai -2a.iv) which is in line with our previous TEM characterisation of PLA-coated Fe_3_O_4_ NPs in a separate study [60,69]. However, NP morphology deviated from spherical when the Fe_3_O_4_ content increased. SEM images of the PLA-coated Fe_3_O_4_ NPs were consistent with the TEM images with a spherical morphology being observed (Fig. S3). When analysing the effect of particle size, the TEM images were also consistent with a prior study in which PEG-coated and uncoated Fe_3_O_4_ NPs (30 nm) (Fig. 2bi and 2b.ii), an average of 29.9 ± 9.8 and 28.3 ± 7.6 nm in diameter was observed, respectively [61]. The morphology of the PEG-coated NPs remained spherical and the NPs were measured to contain 6% w/w PEG (See section 3.1 in Supplementary Information).

**Fig. 3:**
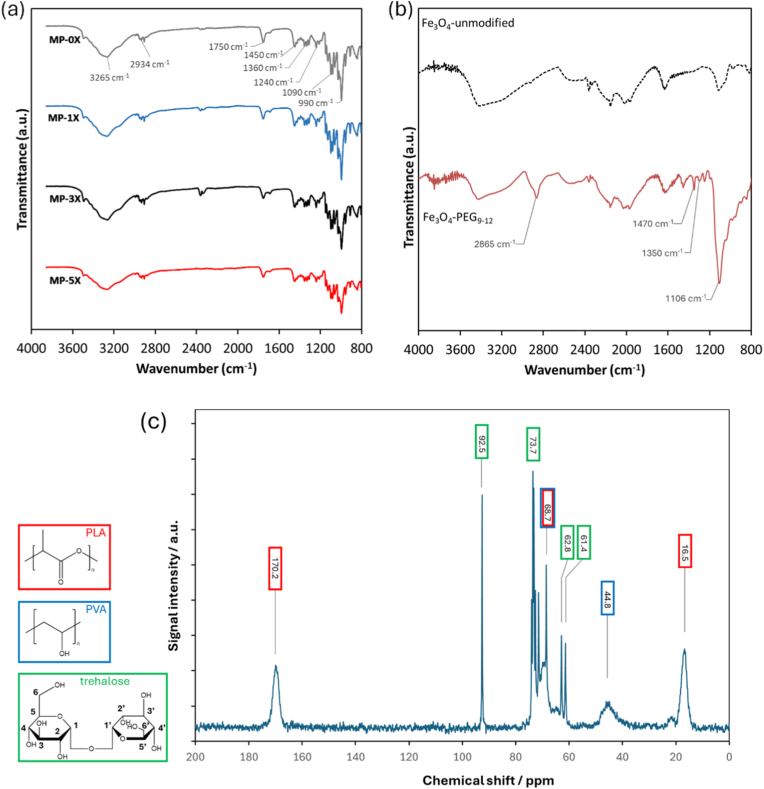
FTIR spectra of (a) PLA-coated NPs and (b) PEG-modified and unmodified Fe_3_O_4_ 30 nm NPs. The solid-state MAS-NMR of MP-0X is shown in (c). The peaks corresponding to the three components as depicted in the colour coding; red: PLA, blue: PVA and green: trehalose.

FTIR was used to study the organic components of the PLA-coated NPs and shown Fig. 3a. The broad band at 3265 cm^−1^ is assigned to the water component in the particle. Evidence of PLA successfully coating the NPs was demonstrated by the presence of strong peaks around 2940 cm^−1^, that are assigned to the symmetric and asymmetric C–H stretching, the characteristic C=O stretching peak at 1750 cm^−1^, as well as bands at 1450 cm^−1^ and 1360 cm^−1^ that are assigned to asymmetric bending of –CH_3_ and –CH–CH_3_, respectively. The complex C–O–C stretching modes of ester groups are also observed between 1240 cm^−1^ and 1090 cm^−1^ [70]. The bands for the trehalose, which was used during the lyophilsation step, are assigned at 1460 cm^−1^ (CH_2_ scissoring) and 1365 – 1312 cm^−1^ (OH deformation), however, these bands are largely shielded by the bands assigned to PLA, as well the band at 990 cm^−1^ assigned to glycosidic bond stretching [71]. The FTIR spectra for uncoated and PEG-coated Fe_3_O_4_ 30 nm NPs is shown in Fig. 3b and the band at 2865 cm^−1^ was attributed to the C–H stretching vibrations while a band a 1470 cm^−1^ was attributed to C–H symmetric bending of a –CH_2_ group. The bands at 1350 cm^−1^ and 1106 cm^−1^ were assigned to the C–O asymmetric bending and the C–O–C stretching, respectively. The FTIR spectra of the samples correspond to the analysis carried out in a previous study [61].

Solid-state MAS NMR analysis of MP-0X also revealed both PLA and trehalose as well as residual PVA (Fig. 3C). The peak at 16.5 ppm was assigned to –CH_3_ while peak at 170.2 was assigned to the CO groups of the PLA structure. The peak at 68.7 was assigned to the CH groups of PLA and residual PVA [72,73]. The –CH_2_ groups from the PVA component was also shown at 44.8 ppm [73]. The rest of the peaks were assigned to the residual trehalose. The peak at 61.4 and 62.6 ppm and was assigned to the CH_2_ group at 6 and 6′ positions while the 73.7 ppm peak was assigned to the CH at 3, 3′ and 5, 5′ positions. The peak at 92.5 ppm was assigned to the CH at 1 and 1’ positions [74]. However, NMR cannot be used to characterise the magnetic nanoparticles studied in this work because of the strong magnetic field that was used in the NMR spectrometer.

### 3.2 DHM control study of live MCF-7 cells

**Fig. 4:**
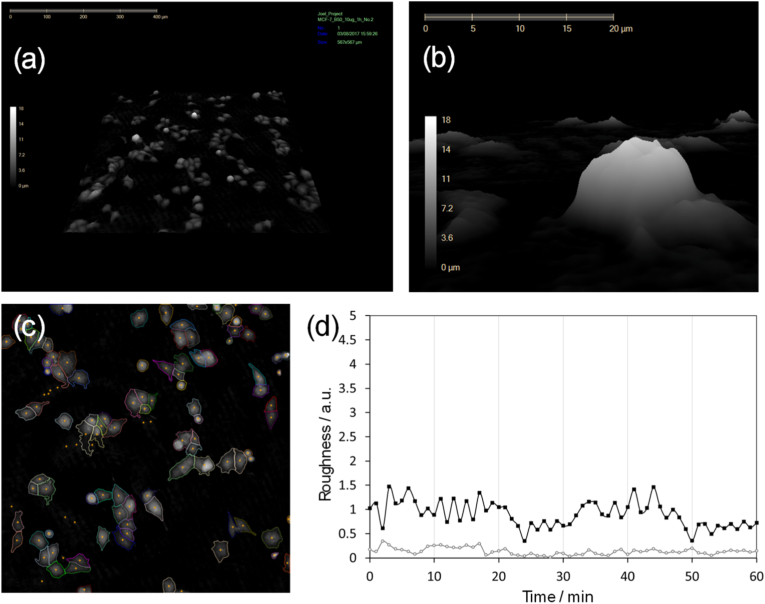
Images from a DHM experiment. (a) An overview 3D map for the captured area, (b) a zoom-in 3-D image of a cell, (c) a 2-D map of the captured area with identified cells, (d) a real time plot of roughness kurtosis (black line) vs roughness skewness (grey line) for the live cell sample.

For DHM analysis, human MCF-7 breast cancer cells were grown and then loaded on to the DHM instrument via a Petri dish. The DHM was set up to capture a small area (1.25 mm × 1.25 mm) of the cell culture and generate a 3-D image of the cell culture (Fig. 4a and b) as well as a 2-D map to identify individual cells (Fig. 4c). To monitor the interaction between cell and NP, cell contour roughness was monitored by using two operational modes of the DHM, kurtosis and skewness that statistically measures the sharpness of peaks and the symmetry of peaks, respectively. From the 2-D map, an individual cell was selected and cell roughness calculated every minute for 1 h. For multi-cell analysis, the average mean value of cell roughness was taken, although abnormal datasets, including cells with unusually high roughness signals, were removed prior to this calculation as this was deemed unlikely to be the result of a cell-NP interaction and disproportionately skewed the final average value. (A detailed procedure is included in the Supplementary Information, Section 2.2). When a foreign object, such as a NP, interacts with the cell surface, an increased signal of roughness will be observed. The fluidised cell membrane will then return to its pre-existing roughness state when the internalisation process is complete, resulting in a reduction in roughness and subsequently a peak when the roughness is plotted against time. When a control experiment was carried out to analyse cell roughness kurtosis and roughness skewness of an MCF-7 cell culture containing no MP-nX, both roughness profiles demonstrated no peaks, signifying that there was no change in roughness of the cell surface over 60 min and the method was appropriate to monitor NP without significant background activity (Fig. 4d). Another control experiment that analysed fixed MCF-7 cells also yielded similar time course graphs containing no peaks, however, the signal-to-noise ratio was dampened as the process of fixing the cells caused the membranes to lose their elasticity [75] (Fig. S6a). Although a small increment on roughness kurtosis was observed when analysing a fixed MCF-7 cell culture incubated with PEG_9-12_-Fe_3_O_4_ NPs, we hypothesised this signal to be NPs accumulating on the cell surface and subsequently Fe_3_O_4_ NP cell internalisation could not be demonstrated using fixed cells (Fig. S6b). These NPs were known to be internalised in various cell types from our prior works [60,61] and here we used fluorescent microscopy and TEM to show NPs were internalised in cells (Fig. S4 and S5).

### 3.3 MP-nX cellular uptake analysis

**Fig. 5:**
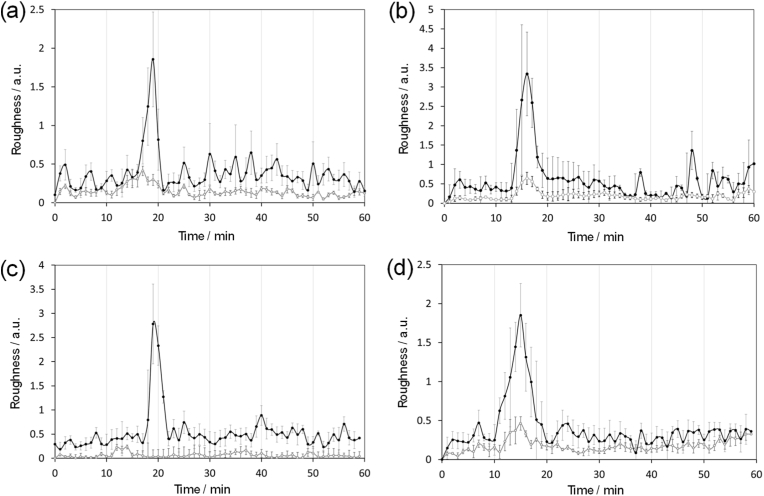
Average plots for roughness kurtosis (black line) and roughness skewness (grey line) of (a) MP-0X, (b) MP-1X, (c) MP-3X and (d) MP-5X. Data = average ± SEM and n = 5.

After carrying out successful control experiments, cellular uptake of the synthesised NPs could subsequently be analysed. The NP with no magnetic content, MP-0X, was initially chosen to determine the appropriate operational mode of DHM to monitor cell roughness. This was carried out by removing the cell culture media from the MCF-7 cancer cells, replacing with serum-free cell culture medium containing MP-0X (5 μg/mL), and then analysing using the DHM instrument. The culture was subsequently incubated at 37 °C with cellular images being collected every minute for 60 min to replicate the conditions of the no NP control. Fortunately, cellular NP uptake was identified in five separate cells, labelled cell 2, 6, 12, 40, and 56, using roughness kurtosis for subsequent analysis. The cells in which NP-cell interactions took place were well distributed (Fig. S7) and when roughness kurtosis was plotted against time a single peak was clearly identifiable for each cell highlighting one discreet internalisation process. It is worth noting that roughness plots for the cells displayed a range of peak heights, which was likely to be due to the different amount of NP interacting with each cell which is difficult to control and, as a result, comparative analysis of peak heights was not included in this study. To improve analysis, the peak start times were normalised in order for the datasets to be compared and the average values of internalisation could be determined. Shown in Fig. 5a, MP-0X absorption to the cell surface initiated on average 15 min after incubation with the cells and demonstrated by an increase in roughness kurtosis. Roughness of the cell surface carried on rising before peaking at 19 min before a sharp decrease was observed indicating that NP internalisation process was complete and the fluidised cell membrane was returning to equilibrium. The peak width indicated the time for initial NP surface absorption to transport into the cell interior took on average 7.2 min. In contrast, NP cellular uptake could not be monitored using roughness skewness as no peak was observed and we hypothesised this was because skewness is a measure of asymmetry of peaks rather than the “sharpness” of peaks, which is more closely related to kurtosis.

After demonstrating that the NP cell internalisation process could be monitored in real-time using MP-0X, NPs with increased magnetite content were then tested to monitor the influence of increasing NP density on cellular uptake. For a direct comparison, the same procedure was carried out as when MP-0X was tested. After incubating MP-1X with MCF-7 cells, five cells were again identified to monitor cellular NP uptake. Similar to MP-0X, a single Kurtosis roughness peak was observed for each cell demonstrating cell internalisation of MP-1X. On further analysis, the peak width was reduced indicating that the rate of cell internalisation was higher, decreasing 1.2-fold from 7.2 to 6.2 min, and suggested that the density of the NP could have an influence on cell internalisation (Fig. 5b). This was further confirmed when MP-3X and MP-5X were tested and a further increase in cellular internalisation rate was observed. As seen in Fig. 5c and d, the average time for cell internalisation for the MP-3X NP was 6.0 min. Conversely, analysis of roughness skewness plots for each NP revealed no observable peak (Fig. 5a–d), indicating that this operational mode is not suitable for monitoring cellular NP uptake.

**Fig. 6:**
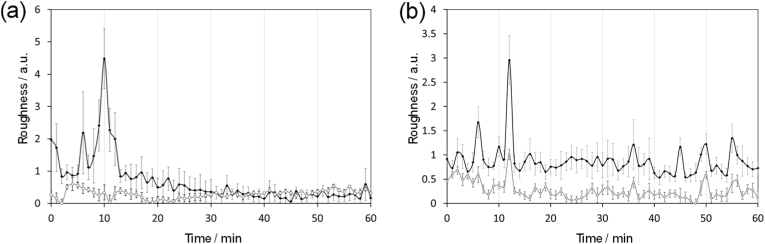
Comparison plots between roughness kurtosis and roughness skewness for (a) Live cells loaded with unfunctionalised Fe_3_O_4_ 30 nm NP and (b) Live cells loaded with PEG_9_-_12_-Fe_3_O_4_ 30 nm NP). Data = average ± SEM and n = 6.

The size of NPs is also a significant factor affecting their uptake mechanism and as a rule of thumb, larger nanoparticles (>250 nm in diameter) will internalise via micropinocytosis while smaller nanoparticles (5-200 nm) will undergo micropinocytosis [76,77]. Therefore, using the same experimental procedure two smaller Fe_3_O_4_ NPs (30 nm) were also tested, one unfunctionalised and one coated with PEG_9-12_. As seen in Fig. 6a, the roughness kurtosis plot for an unfunctionalised Fe_3_O_4_ NPs was similar to that observed from the larger PLA-coated Fe_3_O_4_ NPs. This is possibly a result of unfunctionalised Fe_3_O_4_ NPs tending to form larger aggregates and result in similar physical characteristics to larger NPs. Conversely, smaller PEG_9-12_-coated Fe_3_O_4_ NPs showed a much narrower peak, ca. 2 mins (Fig. 6b), suggesting reduced aggregation and smaller overall particle size increases the rate of cellular NP uptake. This observation is consistent with our previous study and another study that suggests coating Fe_3_O_4_ NPs with PEG reduces NP aggregation [61,78]. Indeed, aggregations can cause inaccuracy in monitoring, generating misleading results as the NPs behaved as larger aggregates, which may alter the uptake mechanism. Therefore, coating the NPs with suitable polymers may be essential to minimise NP aggregation.

## 4 Discussion

### 4.1 Advantages of using DHM for monitoring the NP uptake process

The current state-of-the-art method to monitor NP cellular uptake is using optical microscopy which requires labelling NPs with fluorescent probes and, although useful, there are several disadvantages of using this method *in vitro*. Primarily, fluorescent probes tend to change the surface chemistry of the NP and require the introduction of surface groups to the NP as a covalent linker for probe binding and not all NPs are amenable to the covalent binding of fluorescent probes [43]. In contrast, the DHM method, presented in this work, is label-free technique that can monitor NP interactions with live cells without the requirement of fixing that is commonly required with other techniques, such as fluorescence microscopy to generate good quality images. DHM measures differences in optical path length, or phase shifts, to calculate the optical thickness of the cell by applying Equation 1. The product between actual thickness and the average refractive index, along the optical axis, is then used to reconstruct pseudo-3D images of cells.

$$Phase\;Shift\;\left(\Phi \right)=\left(\frac{2\pi }{\lambda }\right)nx$$

**Equation 1.** Equation to calculate the phase shift of a light wave, where λ is the wavelength of the wave, *n* is the refractive index of the material, and x is the thickness of the material.

As a result, DHM was initially designed to characterise the cell surface and morphology but the short experimental time (circa. 10 mins) and the simple experimental procedure make the technique ideal for studying the cellular uptake of foreign objects in real time [45]. Additionally, simultaneous multi-cell monitoring has also been demonstrated, which is unique to DHM with other techniques taking measurements of single cells. In this study, DHM is used as a label-free kinetic proxy for NP-membrane interaction/internalisation through changes in cell-surface roughness, rather than as a direct substitute for flow cytometry or confocal microscopy/TEM. Flow cytometry remains better suited to population-level endpoint analysis, while confocal microscopy/TEM provides intracellular localisation; by contrast, DHM is complementary in enabling non-invasive, minute-scale time-lapse monitoring under standard culture conditions.

Inspired by Farzam Rad et al., we hypothesised that the use of DHM could be extended to monitoring NP cellular uptake by measuring cell surface roughness over a period of 60 min [55]. We have demonstrated in this study that numerous cells can be monitored simultaneously and averaged to improve the reliability of the results and not relying on a single cell example. Cell roughness was measured by applying the kurtosis and skewness equations to each data point and were compared. After incubating Fe_3_O_4_ NPs with MCF-7 human cancer cells, we observed a change in surface roughness when the NPs interacted with cell membrane that was not observed prior to the interaction and throughout the duration of a negative control experiment without the supplementation of NPs. We hypothesised that when the NP interacted with the cell membrane it caused the cell to appear ‘rougher’ when comparing it to its existing state. Cellular NP internalisation then could be observed with a reduction in cell roughness and a return to the cell membrane pre-existing state of cell roughness. When the skewness equation was applied the background noise was high and any change in cell roughness as a result of the cellular interaction with the NP was not observed and this operational mode could not be applied to monitor NP cellular uptake, in this instance.

Uptake mechanism and rate are also governed by morphology, charges and the surface chemistry of the NP. For example, when analysing cellular uptake of gold nanoparticles (AuNP) with different morphologies, spherical NPs were internalised up to 5 times faster than the equivalent rod-like AuNP when testing STO, HeLa and SNB19 cell lines [79,80]. Additionally, due to the cell membranes negatively charged surface, positively charged NPs exhibit a higher rate of cell internalisation than the neutral and negatively charged NPs, however, are linked to a higher level of cytotoxicity [[81], [82], [83], [84], [85]]. The mechanism of internalisation also changes with positively charged NPs exhibiting a preference for macropinocytosis whereas negative charged NPs tend to internalise via endocytosis [86]. Surface chemistry also plays a significant role in uptake mechanism with hydrophobic NPs embedding within the cell membrane and hydrophilic NPs tend to adsorb to the surface of the membrane and possibly internalised via endocytosis [87,88]. Therefore, developing the understanding of how these variables, as well as other NP parameters including elasticity and NP chemical composition, influence the behaviour of both the cell and the particle is an immediate priority [86]. To this end, we observed a change in NP cellular uptake rate when altering the density of the Fe_3_O_4_ NP as well as when the size. The superior frame rate of DHM enabled roughness kurtosis to be measured at one-minute intervals, allowing us to determine that cellular uptake of Fe₃O₄ NPs increased with increasing density and decreasing particle size. These findings highlight the utility of this technique for monitoring how NP morphology and composition affect cellular uptake and suggest that it may provide an initial basis for elucidating overall NP uptake mechanisms.

### 4.2 Limitations of DHM

Our current method analysed around 5 cells within the image frame (circa. 40 cells in total) taken from each experiment, since we observed that not all cells showed direct NP interaction from NPs in a captured area of 1.558 mm^2^, significantly impacting the dataset. Although a larger n would improve statistical power, this work is a methods-focused proof-of-concept. Only cells with a clear uptake-associated roughness peak were included, while cells with a peak at t = 0 min or cell death before uptake were excluded using predefined criteria. The consistent temporal pattern observed across responding cells supports the reproducibility of the signal, while acknowledging underlying cellular heterogeneity.

In principle, DHM may also support discrimination between viable and non-viable cells by providing continuous, label-free quantitative morphological information during time-lapse imaging. In the present workflow, cells that detached or showed evidence of death before any uptake-associated roughness dynamics were excluded from analysis. However, we do not claim here that DHM replaces established biological viability assays, and dedicated validation against standard live/dead markers would be required for that application. In addition, the speed of internalisation in some instances was quicker than a measurement could be recorded. These limitations cannot be easily controlled experimentally; therefore, a larger sample size or repeated experiments could be applied to improve the reproducibility and reliability of the overall results. It is worth noting that once the cell culture has been loaded with NPs for DHM monitoring, it cannot be reused for another measurement. Therefore, it would be difficult to increase the number of cells (currently around 40) that can be studied in a single experiment. Further work will also seek to apply artificial intelligence and machine learning to reject samples with no change in the roughness signal and to improve the standardisation of peak shifts caused by variation in the time required for NP diffusion to the cell surface.

Diffusion kinetics of NPs is also relevant to drug delivery, however, it is difficult to simulate the conditions *in vivo* using the existing *in vitro* DHM procedure due to the differences in fluid flow, viscosity, and composition of the media (e.g., body fluid vs cell culture medium), as similarly observed in other *in vitro* imaging techniques. Additionally, we cannot state with certainty that all NP samples will behave the same as the selected magnetic NP here due to the limited dataset created in this lone study, therefore, additional studies will need to be carried out to test NP of different chemical compositions. As previously mentioned, various physicochemical properties, such as surface chemistry and particle shape, can affect the mechanism of NP uptake. Despite this study offering some insight into the effects of particle size and density on cellular NP uptake, much more research work is required so we can have a clearer picture of determining the NP uptake mechanism. We will build evidence gained from this proof-of-concept and subsequently study the effect of various experimental parameters, such as cell type, medium type, NP physicochemical characteristics, and NP loading concentrations, as well as extending the workflow to co-culture models (e.g., human liver HepaRG cells) to study nanoparticle-mediated immune-cell targeting, cancer-immune interactions, and cell-cell communication in real time.

## 5 Conclusions

This study reports on the use of digital holographic microscopy (DHM) as a method to monitor nanoparticle (NP) uptake in real time. This non-invasive, label-free technique monitored the cellular uptake of magnetite (Fe_3_O_4_) NPs in multiple MCF-7 human breast cancer cells by measuring cell roughness. This was carried out by applying the kurtosis and skewness equations to measurements from DHM at discrete time points, although kurtosis provided a signal with a higher signal-to-noise ratio than skewness and, therefore, could be used to monitor NP cellular uptake. DHM builds on existing microscopic techniques by allowing us to simultaneously study multiple cells, vastly improving the accuracy of the dataset. Using the average measurement of 5 different cells, we determined that NPs (∼200 nm) took around 4-9 min to be fully internalised, making conventional 3-D confocal microscopy inadequate due to the time required for image processing. For smaller PEG_9-12_-coated Fe_3_O_4_ NPs, the uptake process is even faster (ca. 2 min) due to their smaller size and potentially different uptake mechanism. Building on this case, we believe that this technique can be more widely adopted for the future design of therapeutic NPs, and, more broadly, DHM can be further applied to monitor the cellular NP uptake rate and further mechanistic studies in the future. Future work will focus on applying this technique to various NPs and different types of cell lines. Additionally, we will look to increase the number of cells which are monitored during an experiment to improve the reliability of the data and apply machine learning models to improve the level of accuracy.

## CRediT authorship contribution statement

**Joel Kuhn:** Software, Formal analysis, Data curation, Conceptualization. **Nick W. Johnson:** Writing – review & editing, Writing – original draft. **Christian Curcillo:** Formal analysis. **Lewis A. MacGillivray:** Formal analysis. **Leonard J. Nelson:** Writing – review & editing, Funding acquisition. **Boris Polyak:** Resources, Methodology, Investigation. **Pierre O. Bagnaninchi:** Writing – review & editing, Supervision, Software, Methodology, Investigation, Funding acquisition, Conceptualization. **Humphrey H.P. Yiu:** Writing – review & editing, Writing – original draft, Validation, Project administration, Methodology, Investigation, Formal analysis, Conceptualization.

## Data availability statement

The data presented in this study are available on request from the corresponding author.

## Ethics approval and consent to participate

This study did not involve human participants, human tissue samples, animal experiments, or clinical trials. All experiments were conducted using commercially purchased human breast cancer cell line MCF‑7 (American Type Culture Collection, ATCC, USA), which is an established and widely used cell line in biomedical research without requiring ethical approval for in vitro cell culture studies. Therefore, ethical approval and informed consent were not applicable for this research.

## Funding

This work is supported by the Horizon Europe Halt-RONIN grant #101095679 and UKRI Horizon (Halt-RONIN) Grant #10067052 (L.J.N., P.O.B.). JK also acknowledges the Heriot-Watt University (Fee-only Scholarship), the CareerConcept AG (FESTO Bildungsfonds) and the Deutsche Bildung AG for funding his Ph.D. study.

## Declaration of competing interest

The authors declare the following personal relationships which may be considered as potential competing interests: Boris Polyak is currently employed by Gold Standard Diagnostics Horsham LLC.
