# Supplementary material for: Multi-cell, non-invasive, online monitoring of PLA-coated magnetic nanoparticles uptake by MCF-7 cells using digital holographic microscopy
Source: Bioact Mater. 2026 Apr 16;63:596–606. doi: 10.1016/j.bioactmat.2026.03.053 (PMC13101642; doi:10.1016/j.bioactmat.2026.03.053)
Supplement: Multimedia component 1 [file mmc1.docx]

**Supplementary Information**

**Multi-cell, non-invasive, online monitoring of PLA-coated magnetic nanoparticles uptake by MCF-7 cells using digital holographic microscopy**

Contents

[1. Nanoparticle Synthesis and Characterisation 3](#_Toc224121521)

[1.1. Synthesis of polylactic acid-coated magnetic nanoparticles (MP-0X, MP-1X, MP-3X and MP-5X) 3](#_Toc224121522)

[1.2. Characterisation 3](#_Toc224121523)

[2. DHM data analysis 4](#_Toc224121524)

[2.1. General Information 4](#_Toc224121525)

[2.2. DHM Data Analysis Procedure 6](#_Toc224121526)

[3. Supplementary Data 6](#_Toc224121527)

[3.2. SEM study for MP-nX PLA-coated nanoparticles 7](#_Toc224121532)

[3.3. Fluorescence microscopy of MP-5X-loaded MCF-7 cells 8](#_Toc224121533)

[3.4. Transmission electron microscopy of unfunctionalised Fe_3_O_4_ and PEG_9-12_-Fe_3_O_4_ NPs loaded cells 8](#_Toc224121534)

[3.5. DHM analysis of fixed MCF-7 cells 9](#_Toc224121535)

3.6. DHM analysis of MCF-7 cells loaded with MP-0X particles………………………………9

[4. References 9](#_Toc224121536)

# Nanoparticle Synthesis and Characterisation

# Synthesis of polylactic acid-coated magnetic nanoparticles (MP-0X, MP-1X, MP-3X, and MP-5X)

The procedure general included two steps: i) initial synthesis of oleic acid-coated magnetite (Fe_3_O_4_) fluid, and ii) subsequent formation of MP-nX by coating the prepared Fe_3_O_4_ fluid with polylactic acid (PLA), doped with BODIPY dye. The nomenclature for MP-1X, MP-3X, and MP-5X denotes weight-percent ratios of incorporated magnetite in the polymeric matrix. Magnetite was prepared by alkaline precipitation using the Massart procedure, as reported in ^1^.

**Preparation of the aqueous iron(II)/(III) chloride mixtures:**

**For MP-1X:** Ferric chloride (65 mg, 0.38 mmol, 2 equiv.) and ferrous chloride (24 mg, 0.19 mmol, 1 equiv.) were dissolved in distilled H_2_O (9.0 mL).

**For MP-3X:** Ferric chloride (195 mg, 1.2 mmol, 2 equiv.) and ferrous chloride (72 mg, 0.57 mmol, 1 equiv.) were dissolved in distilled H_2_O (7.1 mL).

**For MP-5X:** Ferric chloride (455 mg, 2.8 mmol, 2 equiv.) and ferrous chloride (168 mg, 1.3 mmol, 1 equiv.) were dissolved in distilled H_2_O (3.3 mL).

In general, sodium hydroxide (1 M, aq) (0.96 mL for MP-1X, 2.9 mL for MP-3X, and 6.7 mL for MP‑5X) was added to the appropriate aqueous mixture of ferric and ferrous chloride to precipitate MP‑nX NPs before being removed from the reaction mixture using a magnet. Each NP was then washed with distilled H_2_O (2 x 10 mL) before being resuspended in EtOH (2 mL) containing oleic acid (100 mg for MP-1X, 150 mg for MP-3X, and 200 mg for MP-5X) and heated, under argon, to 90 °C. After 10 mins, excess oleic acid was removed by phase separation, first adding distilled H_2_O (4 mL) dropwise, then removing the aqueous layer. The organic layer was subsequently washed with EtOH (2 x 10 mL), and the resultant NP was dispersed (28 mg for MP-1X, 84 mg for MP-3X, and 196 mg for MP-5X) in CHCl_3_ (6 mL).

The PLA-coated Fe_3_O_4_ NPs were prepared using the modified emulsification-solvent evaporation method based on the procedure reported in ^2,3^. PLA (200 mg, average M_w_: 75-120 kDa) and PLA fluorescently labelled with BODIPY® (564/570) (20 mg) were dissolved in the appropriate magnetic fluid prepared in the previous section. A cold PVA (15 mL, 1.5% w/v, aq.) solution was added to the organic phase and emulsified by sonication before removing the organic solvent under vacuum. The aqueous solution was filtered using a glass fibre membrane filter (pore size: 1.0 µm) and the resulting particles were lyophilised using trehalose (final concentration: 10% w/v) as a cryoprotectant. The lyophilised NPs were stored at 4°C in 100 µL aliquots and re-suspended in distilled water before use.

## Characterisation

Transmission electron microscopy (TEM) was used to study the morphology of both PLA-coated MP‑nX and the smaller 30 nm Fe_3_O_4_ NP (PEG-coated and unfunctionalized) samples. TEM images of the samples were taken on a Tecnai T20 (FEI) operating at 120 kV (in collaboration with the Kelvin Nanocharacterization Centre, University of Glasgow, UK). In brief, a small amount of the sample (<0.1 μg) was suspended in ethanol and then loaded onto a holy carbon TEM sample grid. Excessive solvent was removed with a clean filter paper, and the loaded grid was air-dried at room temperature. FTIR spectroscopic analysis of both sets of samples was performed using a PerkinElmer Spectrum 100 spectrometer equipped with an attenuated total reflection sampling unit. For the sample measurement, 32 scans over the region 650-4000 cm^-1^ were accumulated (resolution = 4 cm^-1^). The PLA-coated nanoparticle samples (MP-nX series) were imaged by scanning electron microscopy (SEM) with a Quanta 650 FEG SEM in low-vacuum mode (0.83 Torr), at operating voltages of between 5 and 20 kV.

Solid-state magic angle spinning (MAS) NMR spectra were recorded using a Bruker Avance III spectrometer equipped with a wide-bore magnet operating at a magnetic field strength (B0) of 9.4 T (Larmor frequency of 100.6 MHz for ^13^C). Samples were packed into standard ZrO_2_ rotors with an outer diameter of 4 mm and rotated about an axis inclined at the magic angle at a rate of 12.5 kHz using Bruker MAS probes. Chemical shifts are quoted in ppm relative to (CH_3_)_4_Si.

The samples have also been characterised previously in Adams et al^3^.

**1.3 Cell imaging with Nanolive microscope and TEM**

Fluorescence images for MP-5X-loaded live MCF-7 cells were taken using a NanoLive single lens microscope. The STEVE software was used for image acquisition, and the cell culture was kept at 37°C throughout the acquisition. Transmission electron microscopy (TEM) was used to study the HepaRG cells internalised with unfunctionalised Fe_3_O_4_ 30 nm NPs and PEG_9-12_-Fe_3_O_4_ NPs. Details of the method have been published in our prior work^4^.

# DHM data analysis

# General Information

Kurtosis and skewness were determined as the two modes of measurement for roughness in this study. Kurtosis is a statistical measure that describes the distribution of data around the mean and is calculated using Equation S1. The measurement can be used to determine the “peakedness” of a cell surface, with an increased kurtosis signal indicating roughness and appearing as sharper, longer peaks, while a lower kurtosis signal indicates a Bernoulli distribution with shorter, thinner tails.^5,6^ As the experiment searches for peaks, kurtosis can be measured rather than determining the significance of the positive or negative kurtosis values, with a zero value displaying a smooth cell surface.

$$Kurtosis (K)=\sum\frac{{(X_{i}-\bar{X})}^{4}}{{ns}^{4}}$$

**Equation S1.** Equation to determine Kurtosis, where *n* is sample size, $X_{i}$ signifies the *i*th X value, $\bar{X}$ is the average value and *s* is the sample deviation.

The HStudio software, used in this experiment, applies Equation S2 to calculate Kurtosis with a normal distribution around zero to simplify the reference point for comparison.

$$K=\left\{ \frac{n(n+1)}{(n-1)(n-2)(n-3)}\sum\frac{{(X_{i}-\bar{X})}^{4}}{{ns}^{4}} \right\}-\frac{3{(n-1)}^{2}}{(n-2)(n-3)}$$

**Equation S2.** Modified equation to determine Kurtosis with a normal distribution, where *n* is sample size, $X_{i}$ signifies the *i*th X value, $\bar{X}$ is the average value and *s* is the sample deviation.

In contrast, skewness measures the symmetry of data points distributed around the mean by applying Equation S3. The values of roughness are distributed around the mean with zero skew signifying that the values are symmetrical around the mean.^5,6^ . If the skewness value is negative or positive data points are not symmetrical and represents skew and can be used identify a porous or peaked surface.

$$Skewness (S)=\sum\frac{{(X_{i}-\bar{X})}^{3}}{{ns}^{3}}$$

**Equation S3.** Equation to determine Skewness, where *n* is sample size, $X_{i}$ signifies the *i*th X value, $\bar{X}$ is the average value and *s* is the sample deviation.

Similarly to kurtosis, the HStudio software applied a modified version of Equation S3, Equation S4, to calculate skewness to improve accuracy by accounting for sample size. In general, skewness values between 0 to 0.5 are determined as symmetrical, whereas values between 0.5 to 1 are moderately skewed and above 1 is classified as highly skewed.

$$S=\frac{n}{\left( n-1 \right)\left( n-2 \right)}\sum\frac{\left( X_{i}-\bar{X} \right)^{3}}{s^{3}}=\frac{n}{s^{3}\left( n-1 \right)\left( n-2 \right)}\left( S_{above}-S_{below} \right)$$

**Equation S4.** Modified equation to determine Skewness accounting for sample size, where *n* is sample size, $X_{i}$ signifies the *i*th X value, $\bar{X}$ is the average value and *s* is the sample deviation.

**Figure S1.** below illustrates the differences between kurtosis and skewness graphically. A mesokurtic peak depicts a peak that is close or even equal to a normal distribution and here, kurtosis = 3 (excess kurtosis = 0). A sharper peak is described as leptokurtic where kurtosis > 3 (excess kurtosis > 0) while a broader peak is termed as platykurtic as kurtosis < 3 (excess kurtosis < 0). For skewness, a peak near normal distribution will have skewness = 0. Positive skewness of a peak refers to those leaning to the right while those leaning to the left would have a negative skewness.


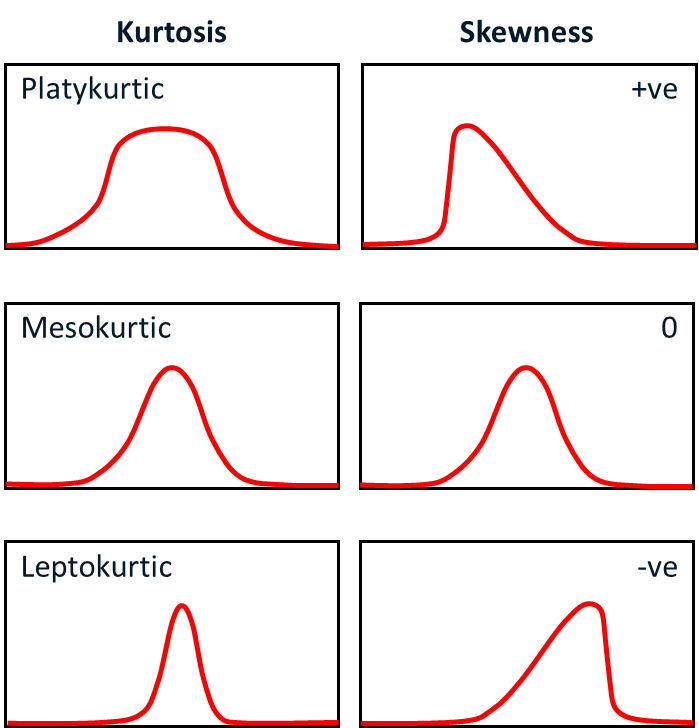


**Figure S1.** Sketches showing the difference between kurtosis and skewness roughness.

## DHM Data Analysis Procedure

Data file was imported into the HStudio software with each experiment analysed individually. To analyse the data from an experiment, all slides was selected from the ‘Track Cells’ tab with blank slides being identified and deleted. To calculate cell kurtosis and skewness, each cell was then selected on the slide and from the ‘Plot Features’ tab, either ‘Cell Kurtosis’ or ‘Cell Skewness’ was selected to apply either Equation S2 or Equation S4, respectively, to each individual cell at each time point. Each cell that demonstrated a kurtosis or skewness signal was then identified. Cells without peaks for either ‘Cell Kurtosis’ or ‘Cell Skewness’ were subsequently discarded and the remaining data displaying roughness kurtosis and skewness was exported to the Origin software for data processing. The procedure was repeated for each experiment.

In order to carry out a multi-cell study with nanoparticles, roughness vs time plots for each cell needed to be plotted out as in Figure S2a. These plots could then be compared and any “abnormality” identified (Figure S2b). For example, cells with unusually high roughness signals over the time period need to be removed because these signals are unlikely to be due to the NP-cell interaction and can significantly alter the average values of the final result. Once all the abnormal data was been removed, the mean value for Roughness Kurtosis/Skewness was calculated (Figure S2c and S2d).

**Figure S2.** Illustration showing the steps for processing the kurtosis data from a multi-cell monitoring experiment using DHM. (a) plotting the kurtosis data of a selected cell over time, (b) plotting out data for all cells, (c) removing the data from cells displaying abnormal behaviour, e.g. cell death, (d) averaging the data with a single plot.

# Supplementary Data

- 1. **Materials characterisation from prior work**

Both set of MNP samples have been published previously in ^3,4^. Table 1 summarises the key properties of PLA-coated MNPs. PEG_9-12_-coated Fe_3_O_4_ NPs showed to have ca. 6% PEG from a thermogravimetric analysis (TGA) against the unfunctionalized sample.

**Table 1.** A summary of the key properties of PLA-coated MNP sample series. Data extracted from^3^.

| PLA-coated MNPs | Magnetite content (estimated from CHN analysis) | Hydrodynamic size of MNPs (from DLS) | ζ-potential | Magnetization at 5 kOe |
| --- | --- | --- | --- | --- |
| MP-0X (non-magnetic) | 0% | 267 ± 0.65 nm | -8.98 ± 0.16 mV | 0 emu/g |
| MP-1X | 6.7% | 262 ± 9.56 nm | -9.46 ± 0.14 mV | 2.11 emu/g |
| MP-3X | 19.2% | 254 ± 2.83 nm | -11.5 ± 0.07 mV | 15.3 emu/g |
| MP-5X | 35.5% | 278 ± 1.62 nm | -14.4 ± 0.34 mV | 24.6 emu/g |


## SEM study for MP-nX PLA-coated nanoparticles


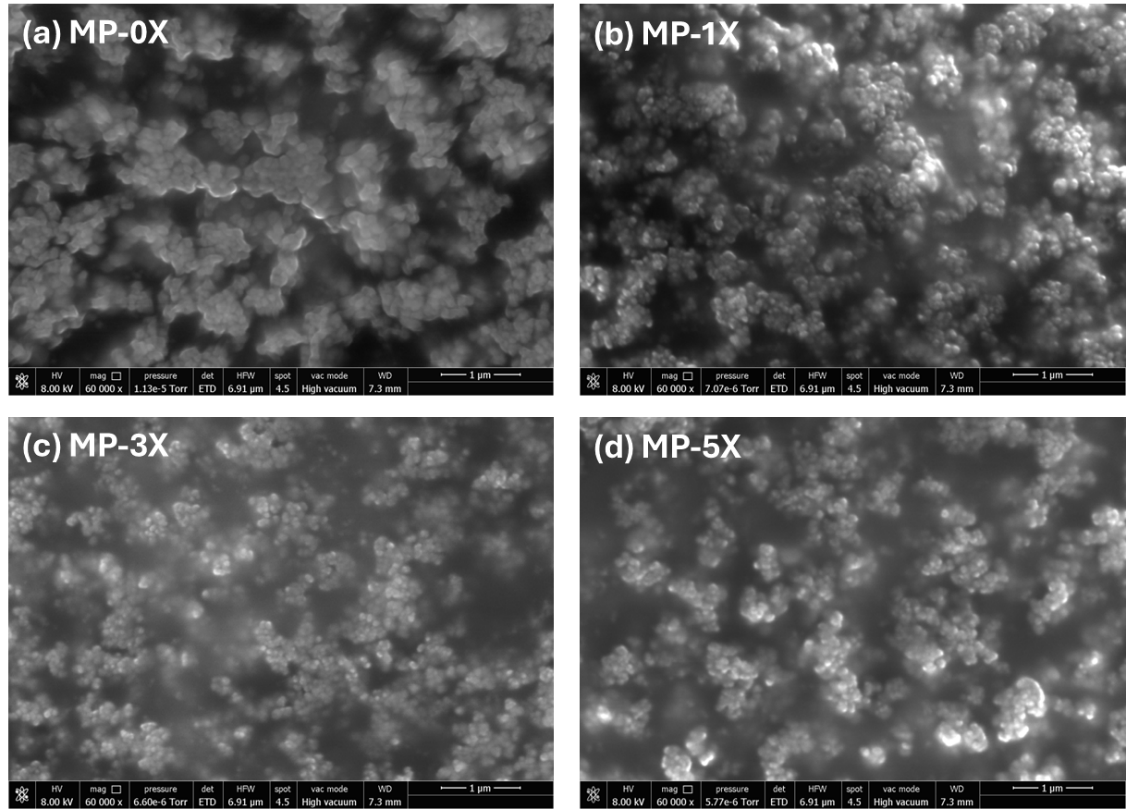


**Figure S3.** SEM images of PLA-coated nanoparticles (a) MP-0X (b) MP-1X (c) MP-3X and (d) MP-5X. Scale bar = 1 μm.

## Fluorescence microscopy of MP-5X-loaded MCF-7 cells


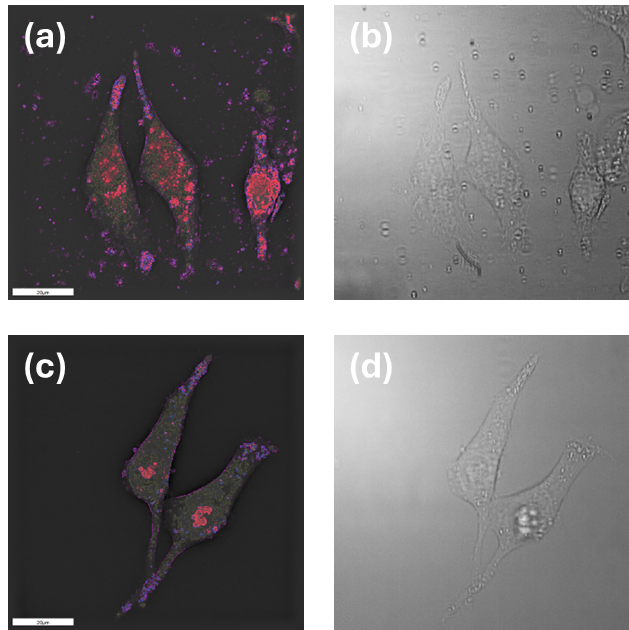


**Figure S4.** Microscopic images of magnetic nanoparticles internalised inside cells. (a) Fluorescence image and (b) bright field image of MP-5X NPs internalised inside MCF-7 cells. The MP-5X NPs were shown as bright red coloured dots. (c) fluorescence and (d) bright field images are control MCF-7 cells with no NPs, showing much cleaner interior in cells. Scale bar = 20 μm.

## Transmission electron microscopy of unfunctionalised Fe3O4 and PEG_9-12_-Fe_3_O_4_ NPs loaded cells


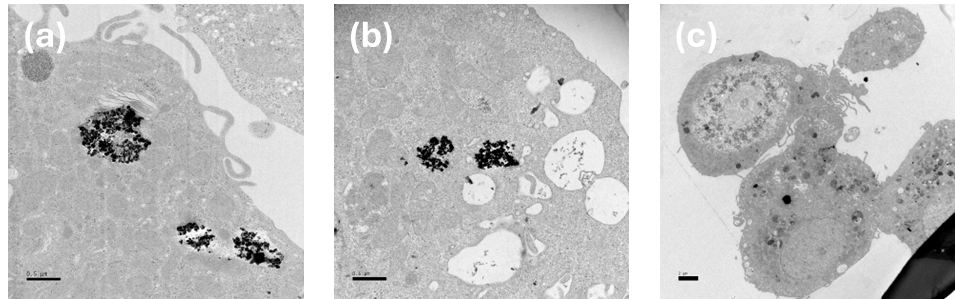


**Figure S5.** TEM image of (a) unfunctionalised Fe_3_O_4_ 30 nm NPs and (b) PEG_9-12_-Fe_3_O_4_ NPs internalised inside HepaRG cells. Scale bar = 500 nm. (c) Controlled experiment of HepaRG cells with no NP loading. Scale bar = 2 μm.

## DHM analysis of fixed MCF-7 cells


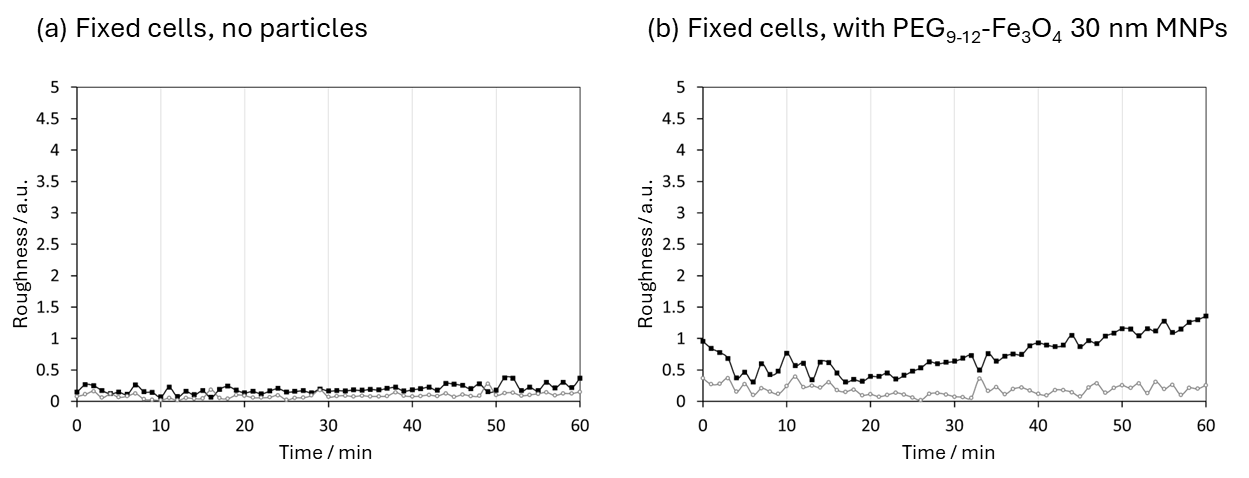


**Figure S6.** Roughness vs time plots for fixed MCF-7 cells (a) without MNPs and (b) loaded with PEG_9-12_-Fe_3_O_4_ 30 nm MNPs

- 1. **DHM analysis of MCF-7 cells loaded with MP-0X particles**

**Figure S7.** Roughness kurtosis plots for MCF-7 cells loaded with MP-0X particles; (a) individual plots for 5 cells that showed signal responding to MP-0X particles and (b) an average of the plots from S6a.

# References

1 R. Massart, Preparation of Aqueous Magnetic Liquids in Alkaline and Acidic Media, *IEEE Trans Magn*, 1981, **17**, 1247–1248.

2 J. A. Tickle, S. I. Jenkins, B. Polyak, M. R. Pickard and D. M. Chari, Endocytotic potential governs magnetic particle loading in dividing neural cells: Studying modes of particle inheritance, *Nanomedicine*, 2016, **11**, 345–358.

3 C. F. Adams, A. Rai, G. Sneddon, H. H. P. Yiu, B. Polyak and D. M. Chari, Increasing magnetite contents of polymeric magnetic particles dramatically improves labeling of neural stem cell transplant populations, *Nanomedicine*, 2015, **11**, 19–29.

4 J. Kuhn, A. McDonald, C. Mongoin, G. Anderson, G. Lafeuillade, S. Mitchell, A. P. D. Elfick, P. O. Bagnaninchi, H. H. P. Yiu and L. J. Nelson, Non-invasive methods of monitoring Fe3O4 magnetic nanoparticle toxicity in human liver HepaRG cells using impedance biosensing and Coherent anti-Stokes Raman spectroscopic (CARS) microscopy, *Toxicol Lett*, 2024, **394**, 92–101.

5 R. Olivares-Navarrete, S. L. Hyzy, M. E. Berg, J. M. Schneider, K. Hotchkiss, Z. Schwartz and B. D. Boyan, Osteoblast Lineage Cells Can Discriminate Microscale Topographic Features on Titanium–Aluminum–Vanadium Surfaces, *Ann Biomed Eng*, 2014, **42**, 2551–2561.

6 Phase Holographic Imaging PHI AB, *HoloMonitor ® M4 Setup and Operation Manual*, Lund, 2018.
